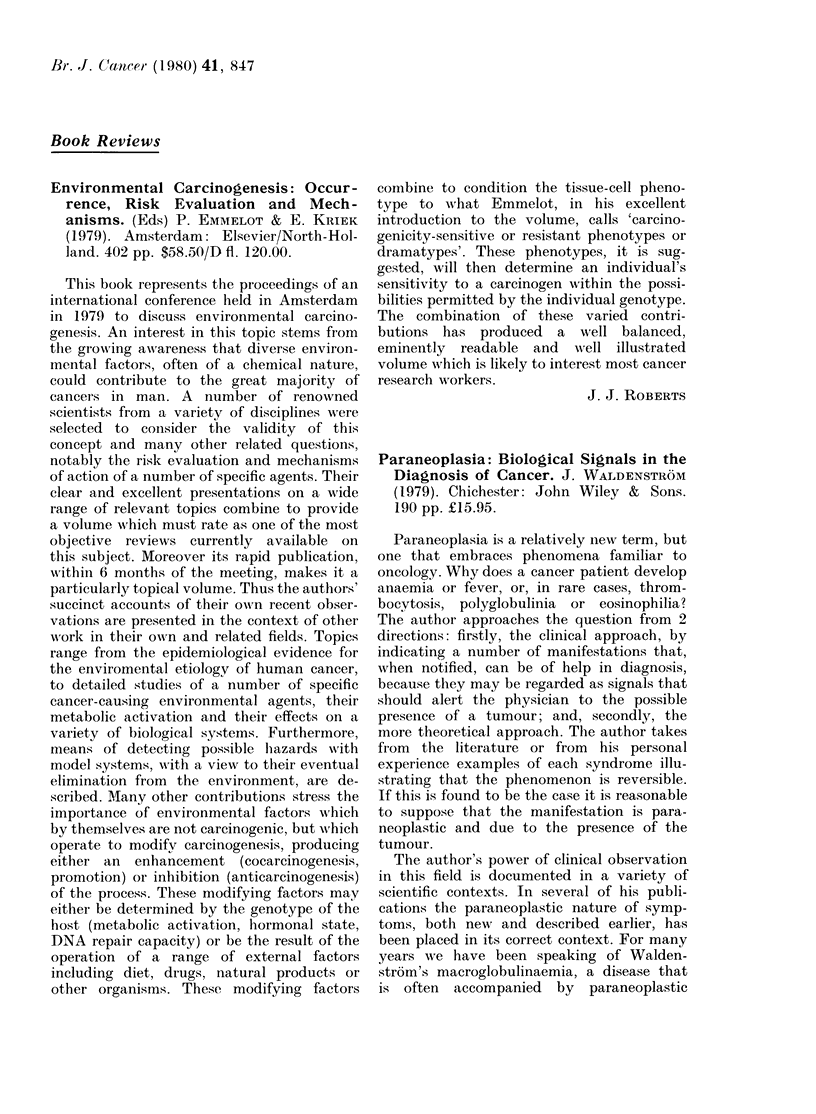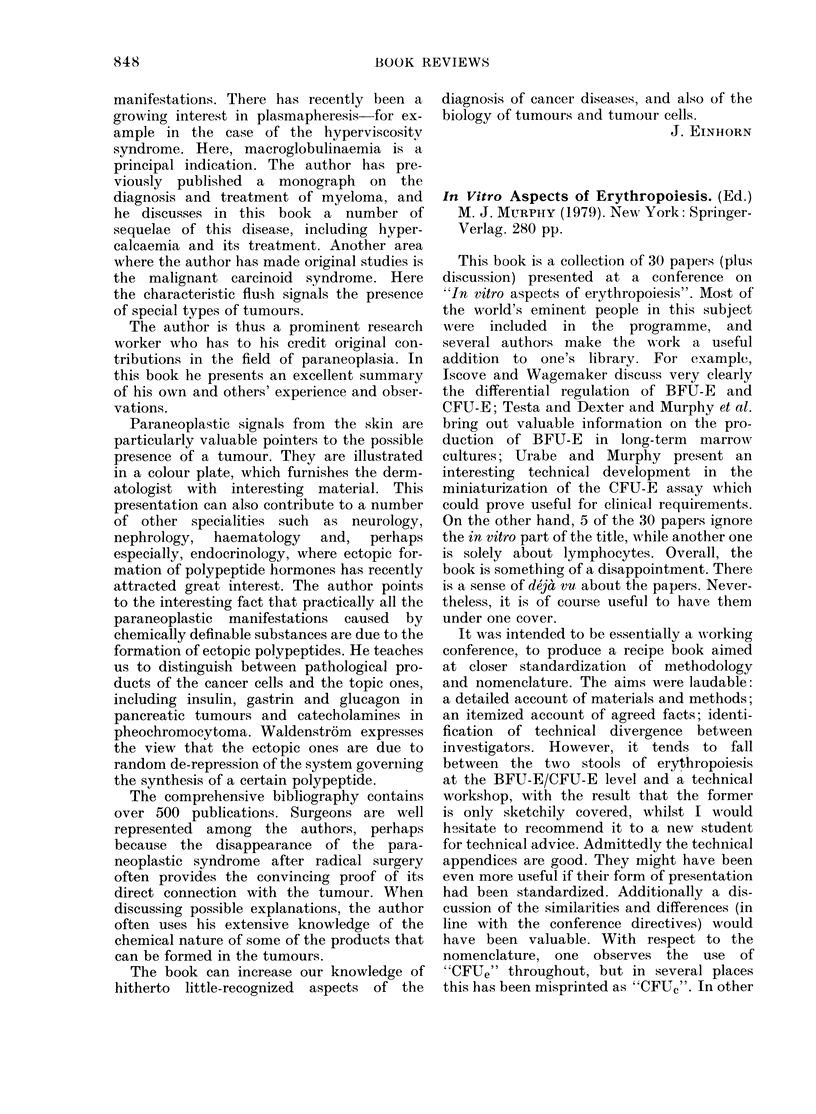# Paraneoplasia: Biological Signals in the Diagnosis of Cancer

**Published:** 1980-05

**Authors:** J. Einhorn


**Paraneoplasia: Biological Signals in the Diagnosis of Cancer.** J. Waldenström (1979). Chichester: John Wiley & Sons. 190 pp. £15.95.

Paraneoplasia is a relatively new term, but one that embraces phenomena familiar to oncology. Why does a cancer patient develop anaemia or fever, or, in rare cases, thrombocytosis, polyglobulinia or eosinophilia? The author approaches the question from 2 directions: firstly, the clinical approach, by indicating a number of manifestations that, when notified, can be of help in diagnosis, because they may be regarded as signals that should alert the physician to the possible presence of a tumour; and, secondly, the more theoretical approach. The author takes from the literature or from his personal experience examples of each syndrome illustrating that the phenomenon is reversible. If this is found to be the case it is reasonable to suppose that the manifestation is paraneoplastic and due to the presence of the tumour.

The author's power of clinical observation in this field is documented in a variety of scientific contexts. In several of his publications the paraneoplastic nature of symptoms, both new and described earlier, has been placed in its correct context. For many years we have been speaking of Waldenström's macroglobulinaemia, a disease that is often accompanied by paraneoplastic manifestations. There has recently been a growing interest in plasmapheresis—for example in the case of the hyperviscosity syndrome. Here, macroglobulinaemia is a principal indication. The author has previously published a monograph on the diagnosis and treatment of myeloma, and he discusses in this book a number of sequelae of this disease, including hypercalcaemia and its treatment. Another area where the author has made original studies is the malignant carcinoid syndrome. Here the characteristic flush signals the presence of special types of tumours.

The author is thus a prominent research worker who has to his credit original contributions in the field of paraneoplasia. In this book he presents an excellent summary of his own and others' experience and observations.

Paraneoplastic signals from the skin are particularly valuable pointers to the possible presence of a tumour. They are illustrated in a colour plate, which furnishes the dermatologist with interesting material. This presentation can also contribute to a number of other specialities such as neurology, nephrology, haematology and, perhaps especially, endocrinology, where ectopic formation of polypeptide hormones has recently attracted great interest. The author points to the interesting fact that practically all the paraneoplastic manifestations caused by chemically definable substances are due to the formation of ectopic polypeptides. He teaches us to distinguish between pathological products of the cancer cells and the topic ones, including insulin, gastrin and glucagon in pancreatic tumours and catecholamines in pheochromocytoma. Waldenström expresses the view that the ectopic ones are due to random de-repression of the system governing the synthesis of a certain polypeptide.

The comprehensive bibliography contains over 500 publications. Surgeons are well represented among the authors, perhaps because the disappearance of the paraneoplastic syndrome after radical surgery often provides the convincing proof of its direct connection with the tumour. When discussing possible explanations, the author often uses his extensive knowledge of the chemical nature of some of the products that can be formed in the tumours.

The book can increase our knowledge of hitherto little-recognized aspects of the diagnosis of cancer diseases, and also of the biology of tumours and tumour cells.

J. Einhorn